# Supplementary material for: Hexane Insoluble Fraction from Purple Rice Extract Retards Carcinogenesis and Castration-Resistant Cancer Growth of Prostate through Suppression of Androgen Receptor Mediated Cell Proliferation and Metabolism
Source: Nutrients. 2020 Feb 20;12(2):558. doi: 10.3390/nu12020558 (PMC7071398; doi:10.3390/nu12020558)
Supplement: Supplementary file 1 [file nutrients-12-00558-s001.pdf]

Figure S1

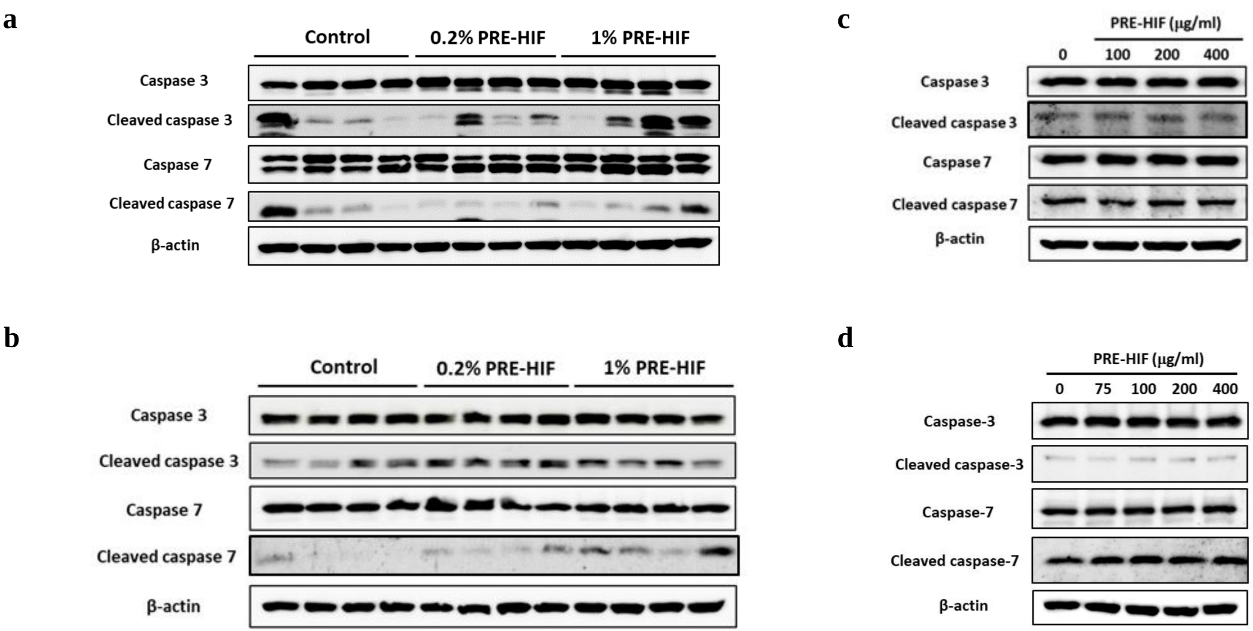

Figure S1 Effects of PRE-HIF on caspase and cleaved caspase expression. Immunoblots of protein lysates from **a**) the lateral prostate samples from TRAP rats, **b**) tumor samples from the PCai1 xenograft model, **c**) PRE-HIF treated LNCaP or **d**) PCai1 cells (72h) were probed with antibodies against proteins related to apoptosis.

Figure S2

a b

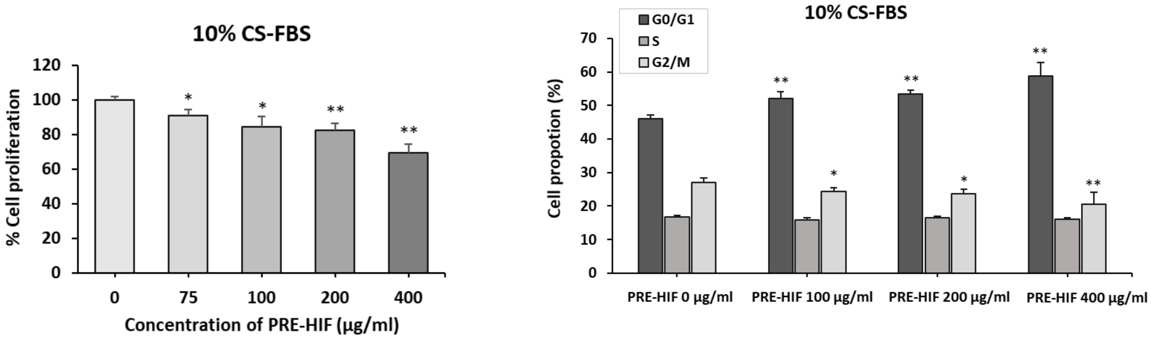

Figure S2 Effects of HIF on rat CRPC cell line PCai1 in completely hormone depleted condition (10% CS-FBS supplemented DMEM). **a**) Cell proliferation of PCai1 cells after incubation with PRE-HIF for 72 hours. **b**) Effects on the cell cycle of PRE-HIF treated PCai1 cells (72h). *p< 0.05;**p< 0.01 compared to control. CS-FBS; Charcoal Stripped Fetal Bovine Serum.
